# Hydrostatic Pressure Induces Osteogenic Differentiation of Single Stem Cells in 3D Viscoelastic Microgels

**DOI:** 10.1002/smsc.202500287

**Published:** 2025-09-21

**Authors:** Nergishan İyisan, Fernando Rangel, Leonard Funke, Bingqiang Pan, Berna Özkale

**Affiliations:** ^1^ Microrobotic Bioengineering Lab (MRBL) School of Computation, Information and Technology Department of Electrical Engineering Technical University of Munich (TUM) Hans‐Piloty‐Straße 1 85748 Garching Germany; ^2^ Munich Institute of Robotics and Machine Intelligence Technical University of Munich Georg‐Brauchle‐Ring 60 80992 München Germany; ^3^ Munich Institute of Biomedical Engineering Technical University of Munich Boltzmannstraße 11 85748 Garching Germany

**Keywords:** hydrostatic pressure, mechanotransduction, mesenchymal stem cells, microfluidic cell encapsulation, microgels, osteogenic differentiation

## Abstract

Sustained mechanical stimulation represents a powerful strategy for directing stem cell fate, yet its application within microscale injectable carriers remains limited. This study presents a dynamic microgel platform enabling osteogenic differentiation of single mesenchymal stem cells (MSCs) solely through hydrostatic pressure, without biochemical induction. Individual MSCs are encapsulated in ionically crosslinked, cell‐adhesive alginate microgels and stabilized using an alginate–poly‐l‐lysine–alginate and calcium coating. Application of cyclic hydrostatic pressure at 200 kPa and 0.5 Hz frequency for 30 min per day leads to upregulation of early osteogenic markers RUNX2 and alkaline phosphatase, enhanced collagen I synthesis, and mineralization over 21 days. Results demonstrate that mechanical cues alone are sufficient to orchestrate osteogenic commitment in soft, confined microenvironments, offering a scalable approach to stem cell programming. This work establishes a versatile, high‐resolution platform for engineering lineage specification at the single‐cell level and highlights the potential of force‐driven strategies for scalable production of therapeutic stem cells.

## Graphical abstract

Single mesenchymal stem cells are encapsulated in arginine‐glycine‐aspartic acid‐functionalized alginate microgels using a microfluidic chip. After secondary crosslinking, cells are cultured under cyclic hydrostatic pressure in a custom pressurization setup. Collagen type I expression increases over time under dynamic conditions compared to static controls, indicating mechanical stimulation alone drives osteogenic differentiation within soft, confined 3D microenvironments.

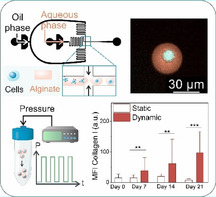

## 1 Introduction

Mesenchymal stem cells (MSCs) have gained widespread interest in regenerative medicine due to their ability to differentiate into multiple lineages and to secrete paracrine factors that promote angiogenesis, modulate inflammation, and support tissue repair.^[^ ^1^, ^2^, ^3^, ^4^ ^]^ The use of biomaterials as supportive scaffolds has improved the therapeutic use of MSCs by addressing challenges such as poor cell retention, survival, and integration at target sites^[^ ^5^, ^6^ ^]^ while providing mechanical support for soft and hard tissue defects and promoting vascularization.^[^ ^7^, ^8^, ^9^ ^]^ Owing to their tunable biochemical and mechanical properties, hydrogels can be engineered into effective cell carrier systems that not only support MSC survival and retention but also guide their fate through controlled microenvironmental cues.^[^ ^10^, ^11^ ^]^

Building on these advances, microgels have emerged as tunable and modular cell carrier platforms for stem cell delivery and manipulation. Various strategies have been employed to regulate stem cell behavior within microgels, including biochemical functionalization, mechanical tuning, and geometric confinement.^[^ ^12^, ^13^, ^14^, ^15^, ^16^, ^17^, ^18^ ^]^ Among these approaches, the use of stimuli‐responsive microgels remains the main method to trigger the differentiation of encapsulated adult stem cells by releasing biochemical cues in a spatially confined manner.^[^ ^14^ ^]^ Despite these advances, current strategies heavily rely on biochemical induction, which lacks physiological relevance and poses translational hurdles such as batch‐to‐batch variability, off‐target effects, and safety concerns. Existing microgel platforms often remain static and are not easily integrated into dynamic, scalable workflows for seamless processing. These limitations highlight the need for mechanically active systems capable of directing stem cell fate through dynamic biomechanical cues.

Mechanical cues in the form of cyclic tension, shear stress, and hydrostatic pressure have been shown to induce stem cell differentiation at larger scales,^[^ ^19^, ^20^ ^]^ but the implementation of these principles to microscale carriers has proven difficult. It has only recently been possible to demonstrate short‐term mechanical cueing in microenvironments at this scale using micromanipulation and nanorobotic tools.^[^ ^21^, ^22^, ^23^ ^]^ In particular, applying sustained mechanical stimulation to singly encapsulated stem cells within soft, injectable microgels remain underexplored. This feature is especially critical during differentiation, as encapsulating cells after lineage commitment risks disrupting fragile intermediate states and losing responsiveness to mechanical signals.^[^ ^24^, ^25^ ^]^ Preparing stem cells within microgels from the beginning not only protects their native mechanosensitivity but also allows their differentiation to proceed within a stable microenvironment, eliminating the need for postprocessing or adaptation.^[^ ^24^, ^25^ ^]^ A seamless cell preparation platform would thus combine early packaging, long‐term culture, and dynamic stimulation to guide cell fate efficiently and reproducibly.

**Figure 1:**
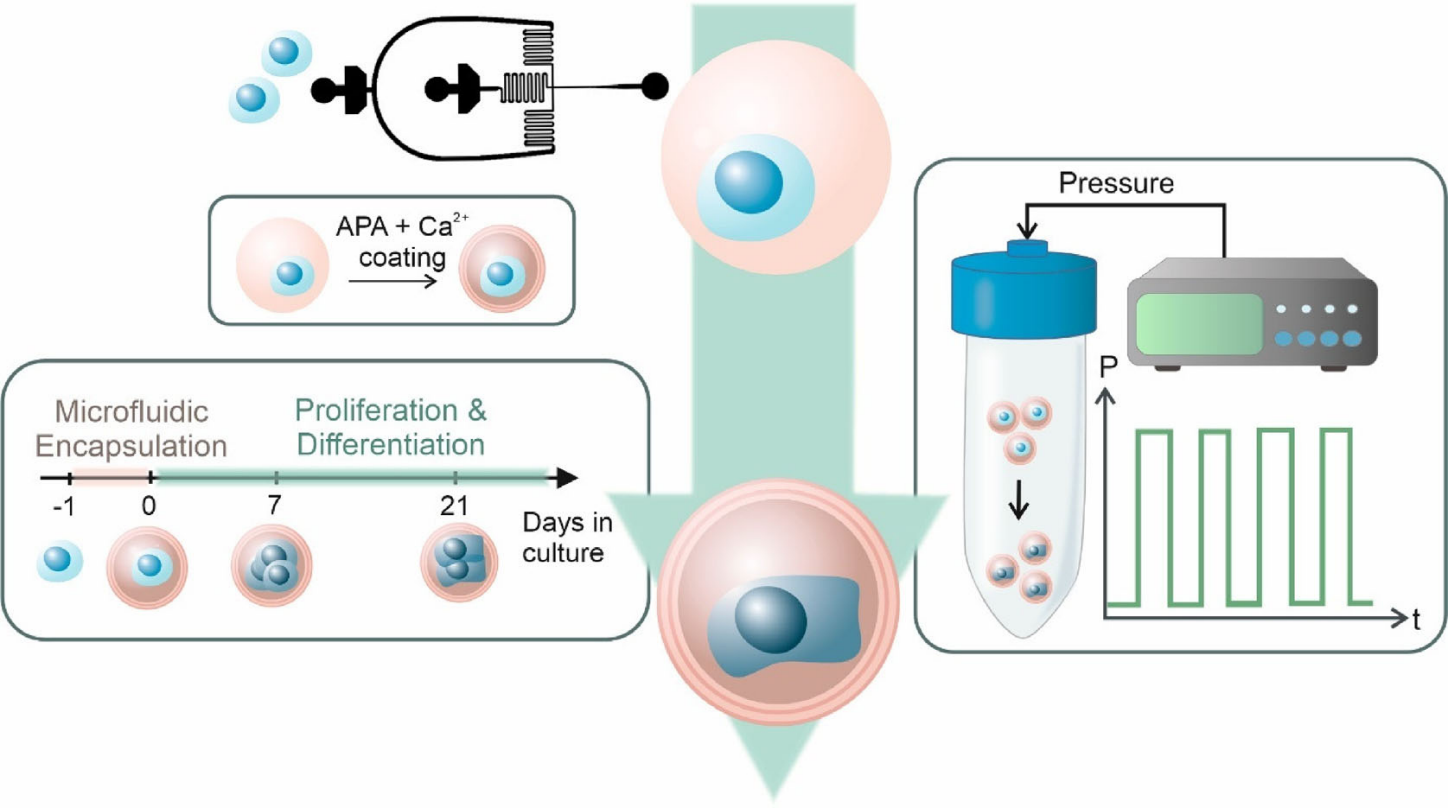
Schematic overview of the experimental workflow. MSCs (blue) were singly encapsulated using a microfluidic approach within calcium‐crosslinked, RGD‐functionalized alginate microgels (pink), followed by a secondary APA and calcium coating to enhance stability. Encapsulated cells were cultured for 21 days and subjected to cyclic hydrostatic pressure in regular cell culture media without any growth factors.

To address this challenge, we engineered a mechanically active cell culture platform relying on hydrostatic pressure to induce the differentiation of singly encapsulated MSCs toward osteogenesis within soft and viscoelastic 3D alginate microgels (≈1.5 kPa in culture media) (**Figure** **1**). We utilized microfluidics to encapsulate individual MSCs within ionically crosslinked, arginine‐glycine‐aspartic acid (RGD)‐functionalized alginate microgels and stabilized them through secondary alginate–poly‐l‐lysine–alginate (APA) and calcium coating. We previously demonstrated the feasibility of pressure‐actuated alginate microgels for delivering defined mechanical cues to single MSCs, generating sufficient pressure to trigger mechanotransduction at the microscale.^[^ ^26^ ^]^ Based on this, we applied cyclic hydrostatic pressure (200 kPa, 0.5 Hz) over 3 weeks in the absence of osteogenic induction media. Pressure‐stimulated singly encapsulated cells exhibited increased nuclear Yes‐associated protein (YAP) localization, followed by elevated expression of early osteogenic markers RUNX2 and alkaline phosphatase (ALP). Continued stimulation led to elevated collagen I and laminin synthesis. These results demonstrate that cyclic mechanical cues alone are sufficient to induce osteogenic differentiation in soft, viscoelastic single‐cell microgels without induction media, thereby overcoming the need for stiffer matrices and offering a scalable strategy for guiding stem cell fate at single‐cell resolution.

## 2 Results and Discussion

### 2.1 Microfluidic Encapsulation and Stabilization of Single MSCs in Alginate Microgels

**Figure 2:**
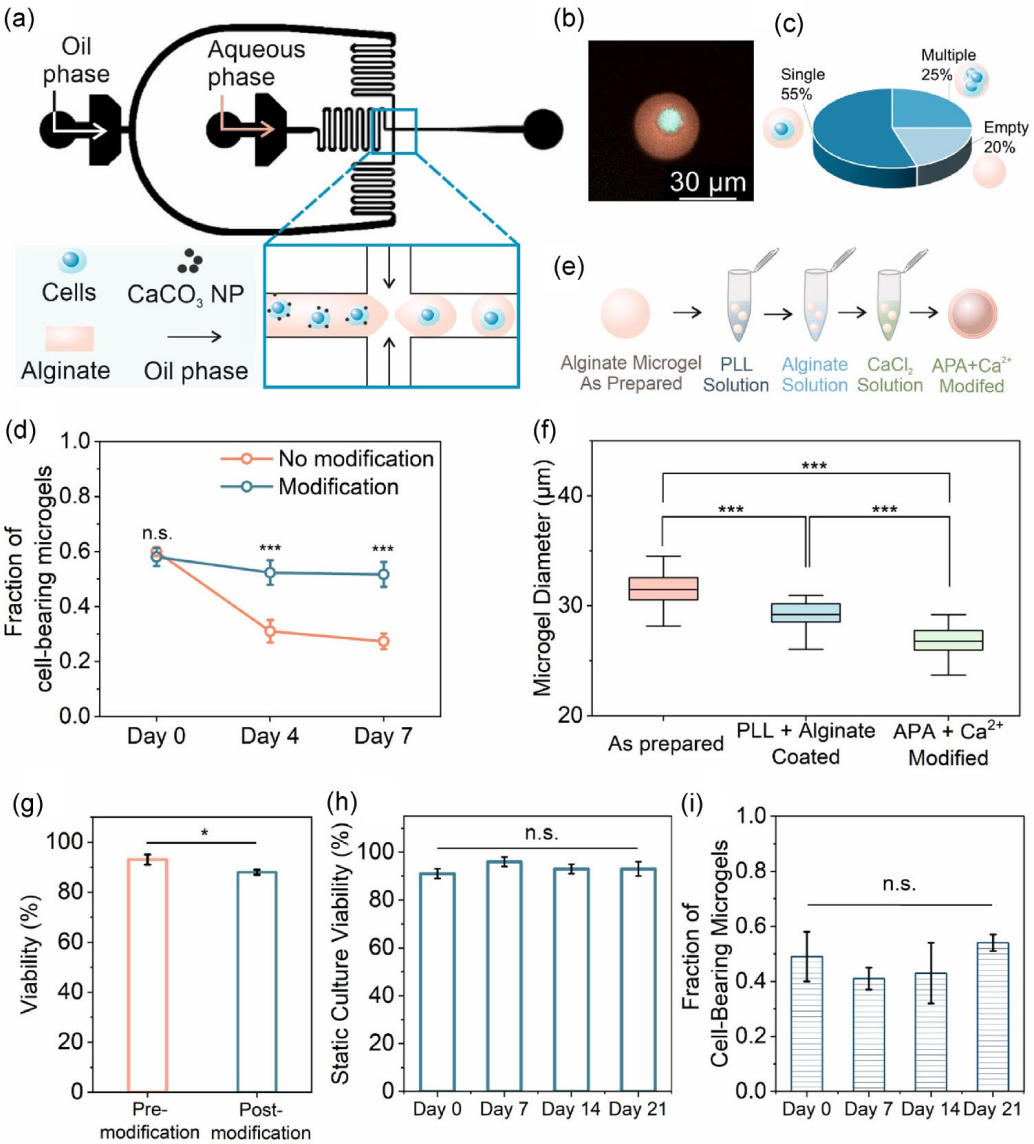
Microfluidic encapsulation of MSCs and stabilization of alginate microgels for long‐term culture. a) Schematic representation of the microfluidic encapsulation process. MSCs pretreated with calcium carbonate nanoparticles (CaCO_3_ NPs) were suspended in RGD‐functionalized alginate and introduced into the aqueous phase. Fluorinated oil containing 0.04 vol% acetic acid and surfactant served as the continuous phase. Upon droplet formation, acetic acid diffused into the droplets, dissolving CaCO_3_ NPs and releasing calcium ions to crosslink the cell‐loaded alginate droplets. b) A representative image of a single MSC encapsulated in an alginate microgel. Rhodamine B fluorescence (pink) indicates the alginate matrix, while Calcein AM signal (cyan) marks the live MSC. c) Distribution of encapsulation outcomes showing that 55% of microgels contained a single MSC, while 25% contained multiple cells and 20% were empty (*n* = 100). d) The fraction of cell‐bearing microgels significantly declined over time in unmodified samples (pink line), whereas coated microgels (blue line) retained cells over 7 days (*n* = 100, three replicates, Student's *t*‐test). e) Schematic of the post‐encapsulation APA and calcium modification process. f) Microgel diameter decreased after each modification step, confirming successful coating and compaction (*n* = 30, one‐way ANOVA followed by Tukey's post hoc test). g) Cell viability before and after APA modification showed a slight but statistically significant decrease after APA+Ca^2+^ modification (*n* = 100, three replicates, Student's *t*‐test). h) Long‐term viability of encapsulated cells is presented, which remained high over 21 days of static culture (*n* = 100, three replicates, one‐way ANOVA). i) The fraction of cell‐bearing microgels measured over three weeks under static culture conditions (*n* = 100, three replicates, one‐way ANOVA). n.s. indicates not significant; *p < 0.05, **p < 0.01, ***p < 0.001. Error bars represent standard deviation.

In this work, our primary goal was to induce osteogenic differentiation of singly encapsulated MSCs through mechanical stimulation alone, without the use of osteogenic induction media. To enable differentiation within microgels, it was first necessary to establish long‐term culture conditions that would support cell viability and structural confinement of the encapsulated MSCs over 3 weeks. For this purpose, we adapted a previously developed approach,^[^ ^26^ ^]^ specifically using microfluidics to encapsulate them within calcium‐crosslinked, RGD‐functionalized alginate hydrogels (**Figure** **2**a). Cells were pre‐incubated with CaCO_3_ nanoparticles and encapsulated in RGD‐modified alginate using a microfluidic device, where acetic acid in the oil phase triggered CaCO_3_ dissolution in droplets and subsequent crosslinking of alginate, yielding cell‐laden microgels upon demulsification and washing (Figure 2b). With this approach, MSCs were encapsulated in alginate microgels, with the majority of the microgels (55%) containing a single MSC (Figure 2c). However, maintaining the cells in long‐term culture within the microgels presented some challenges. Cell egression was observed within 4 days of encapsulation, likely due to continued cell viability, proliferation, and matrix remodeling, which led to cell eggresion from the microgels and led to a marked decrease in the fraction of cell‐laden microgels (Figure 2d).

To resolve this issue, microgels were treated with a secondary alginate coating and calcium treatment immediately following cell encapsulation to improve the structural integrity of the microgels (Figure 2e). Briefly, microgels were first treated with the cationic polymer poly‐l‐lysine (PLL), followed by treatment in an un‐crosslinked alginate solution (0.001%). This approach leads to the formation of a secondary layer of alginate over the entire surface of the microgels due to the strong electrostatic interaction between negatively charged alginate chains and the positively charged PLL.^[^ ^27^ ^]^ Subjecting the doubly coated microgels to a low concentration of (30 mM) calcium chloride allowed crosslinking the secondary alginate shell, which reinforced microgel stability.^[^ ^28^, ^29^ ^]^ Microgel size was measured before and immediately after each modification to confirm the presence of the secondary layers, as both PLL and calcium treatment are known to reduce alginate microgel size.^[^ ^29^ ^]^ PLL reduces microgel size through the strong electrostatic interaction between positively charged PLL and negatively charged alginate,^[^ ^27^ ^]^ while the subsequent calcium treatment provides an additional crosslinking between the alginate, leading to further compaction in treated microgels.^[^ ^27^ ^]^ As expected, the alginate microgels decreased in size from ≈32 ± 1.5 μm to 29 ± 1.6 μm following the PLL‐alginate treatment and further reduced to around 27 ± 1.6 μm after the calcium treatment (Figure 2f). A significant difference was observed between modified and unmodified microgels, with the fraction of cell‐bearing microgels remaining significantly higher in the modified group at both day 4 and day 7 (Figure 2d). Within the modified group, the fraction of cell‐bearing microgels remained stable over time between day 0 and day 7, indicating that the modification successfully prevented further cell egression. This step was essential in supporting our overall objective, ensuring that the cells remained within their 3D culture environment throughout the differentiation process, and it provided a stable platform for subsequent experiments.

We evaluated cell viability to confirm that the modification process did not compromise cell health. Encapsulated cell viability remained high at ≈90% in unmodified and PLL‐alginate modified microgels, with only a slight decrease from ≈93% to 87% after microgel modification (Figure 2g). While this reduction was statistically significant, it was not biologically relevant as the majority of the cells remained viable. The minor decrease in viability was likely due to the time cells spent outside the incubator during multiple centrifugation steps. Long‐term viability over 21 days remained stable, with no significant differences between days 0, 7, 14, and 21, confirming that the microgel environment supported long‐term cell survival (Figure 2h). Encapsulated cell analysis showed there was no significant difference in the number of cell‐bearing microgels from day 0 to day 21 (Figure 2i), further supporting the effectiveness of the encapsulated MSC culture method in maintaining encapsulated and viable cells throughout three weeks.

### 2.2 Long‐Term Pressure Application and Mechanosensitive Response of Encapsulated MSCs

To enable mechanical actuation of encapsulated cells, we designed a platform based on three core design criteria: 1) the ability to maintain encapsulated MSCs in microgels under long‐term culture conditions for 3 weeks; 2) reliable delivery of hydrostatic pressure with tunable parameters such as amplitude, frequency, duty cycle, and duration; and 3) a robust and easily reproducible mechanical setup. To fulfill these requirements, we focused on modifying standard cell culture vessels with sealed ports to allow controlled pressurization.

The primary configuration employed modified conical tubes, which offered a simple and scalable format compatible with long‐term culture of encapsulated cells. In addition, sealed well plates were used for a subset of 2D monolayer experiments as control. These two formats, represented schematically by solid lines (conical tubes) and dotted lines (well plates) (Figure S1a, Supporting Information), enabled consistent hydrostatic pressure delivery across different experimental needs while maintaining sterility, ease of use, and compatibility with standard lab infrastructure. Here, we adapted our previous pressure‐stimulation system^[^ ^26^ ^]^ to a larger setup and quantified how headspace volume affects pressure dynamics. In the earlier chamber, minimal headspace allowed near‐instantaneous pressure transfer, but larger vessels introduce delays that can distort a 0.5 Hz waveform. To address this, we characterized the delay using an inline sensor before biological experiments. Comparing 15 and 50 mL tubes with equal headspace showed that rise time depends on headspace, not vessel volume (Figure S1b, Supporting Information). Systematic variation of headspace (15–55 mL) revealed a linear dependence of rise time on headspace volume (Figure S1c, Supporting Information). This calibration enables us to preserve waveform amplitude and frequency across different vessels. Guided by this, we used a 15 mL conical tube as the pressure vessel and established a sterile, airtight system (Figure S1d, Supporting Information). A standard 15 mL conical tube with a custom‐modified cap containing a central aperture was connected to the pressure controller via rigid plastic extension tubing. Two wide‐bore syringe filters were incorporated in series to ensure sterile delivery of compressed air to the vessel. All junctions were secured with Luer‐lock couplings, creating a rigid, dimension‐matched assembly with close‐fitting screw‐type connections that ensured mechanical stability, an airtight seal, and sustained pressure integrity throughout repeated pressurization cycles (Figure S1d,e, Supporting Information).

Uniform pressure distribution within the culture medium is achieved due to the incompressibility of the liquid, whereby any externally applied pressure to a confined fluid is transmitted equally in all directions. Given that the culture medium is nearly incompressible, the air pressure applied to the headspace propagates almost instantaneously and uniformly throughout the liquid, allowing the assumption that all microgels are subjected to the same pressure amplitude to be accurate. We chose a hydrostatic pressure value of 200 kPa, which has been reported to induce osteogenic differentiation of adult stem cells in vitro.^[^ ^20^ ^]^ A frequency range of 0.5–2 Hz was selected due to its biological relevance, simulating typical human gait frequencies^[^ ^30^ ^]^ and leading to osteogenesis in MSCs in pressurized cell culture systems.^[^ ^31^ ^]^ Both the well‐plate and conical tube pressure devices succeeded in applying pressure at 200 kPa at 0.5 and 1 Hz. However, a significant reduction in the output pressure was recorded at a frequency of 2 Hz (Figure S2, Supporting Information). This issue likely stems from the larger volume of the conical tube with respect to the well plate, which requires a greater amount of air to reach 200 kPa in a shorter amount of time at 2 Hz compared to lower frequencies. As a result, the response to high‐frequency pressure application is slower. Despite the decrease in pressure at higher frequencies, both pressure devices remained effective at 200 kPa and 0.5 and 1 Hz, confirming their suitability for proceeding with encapsulated MSC differentiation experiments.

**Figure 3:**
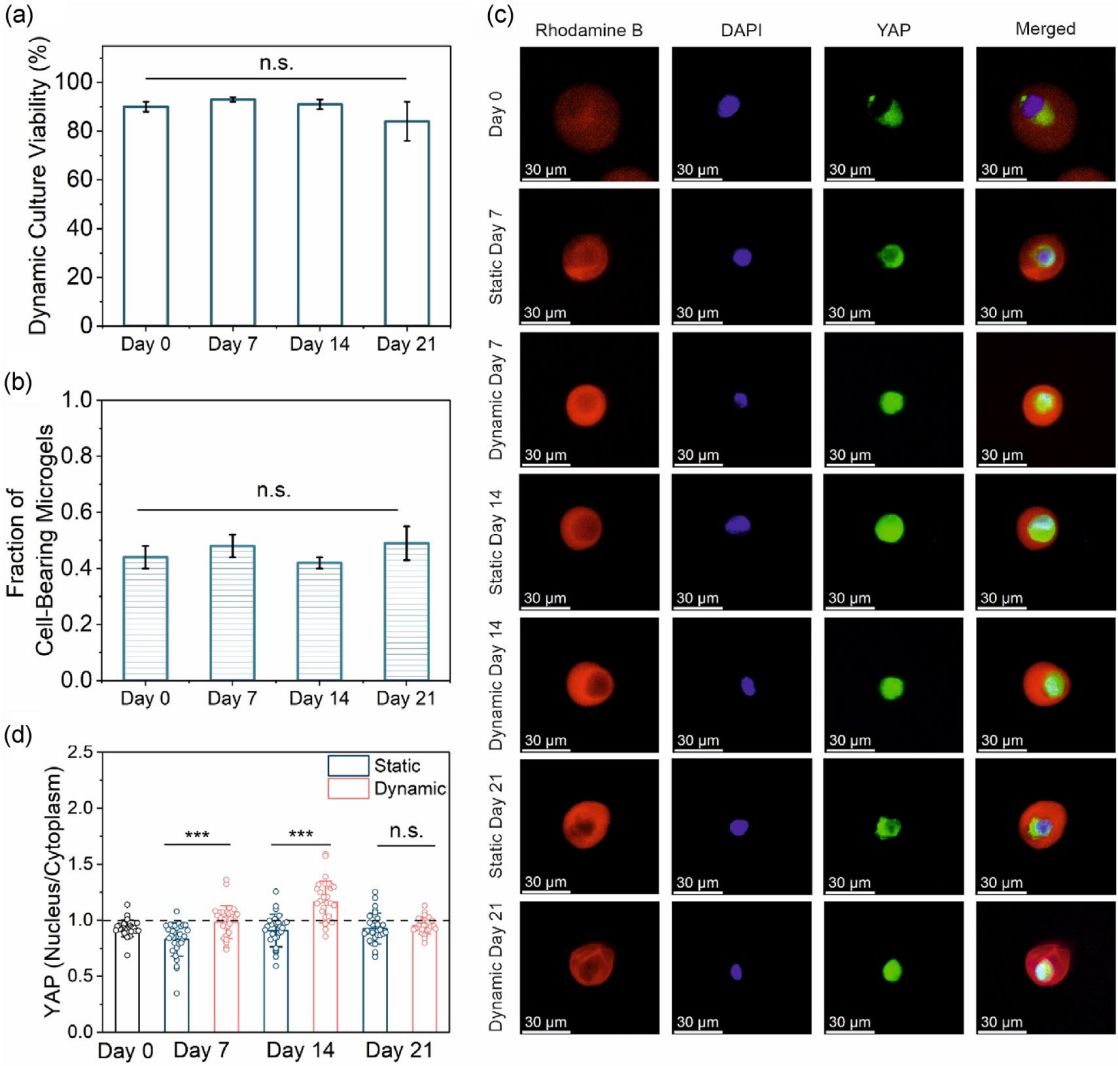
Evaluation of encapsulated MSC response to long‐term dynamic mechanical stimulation in microgels. a) Cell viability of encapsulated MSCs cultured under dynamic culture conditions (*n* = 100, three replicates, one‐way ANOVA). b) The fraction of microgels retaining encapsulated cells throughout the three‐week dynamic culture period (*n* = 100, three replicates, one‐way ANOVA). c) Representative fluorescence microscopy images showing YAP localization in encapsulated MSCs over time. Rhodamine B (red) marks the microgel boundary, DAPI (blue) indicates the nucleus, and YAP (green) visualizes subcellular distribution. d) Quantification of the nuclear‐to‐cytoplasmic YAP fluorescence intensity ratio (*n* = 30 per group, Student's *t*‐test). n.s. indicates not significant; *p < 0.05, **p < 0.01, ***p < 0.001. Error bars represent standard deviation.

We next investigated the long‐term effects of pressure on encapsulated cells. The conical tube pressure device was used to apply 200 kPa pressure on encapsulated MSCs at 0.5 Hz frequency (50% duty cycle) for 30 min over 21 days. It was crucial to confirm that the pressure‐exposed cells remained viable over the 3 week period, as this duration is necessary for osteogenic differentiation. The encapsulated cells maintained a viability of ≈90% from day 0 to day 14, with a slight decrease to around 80% on day 21. However, this reduction was not statistically significant across the measured time points, indicating that the applied pressure did not negatively impact long‐term cell viability (**Figure** **3**a). We also confirmed that the cells remained within their 3D microenvironments throughout the pressure application process, as the fraction of cell‐bearing microgels remained stable over time with no significant changes detected (Figure 3b). Cell proliferation within the 30 μm microgels remained minimal over the 3 week period, with no significant difference compared to the nonpressurized control group (Figure S3, Supporting Information). The confined structure likely restricted proliferation, consistent with observations by Mao et al. in cells experiencing spatial constraints.^[^ ^29^ ^]^ Limited proliferation is desirable here, enabling analysis of single‐cell responses to pressure while minimizing cell‐to‐cell signaling and reducing differentiation heterogeneity.^[^ ^32^ ^]^ Cell size remained comparable between dynamic and static conditions, with no significant differences observed (Figure S4, Supporting Information). However, a statistically significant increase in nucleus diameter was observed in the dynamic culture group compared to the static one. These findings suggest that while overall cell size was not affected by pressure application, nuclear expansion may be a response to mechanical stimulation and differentiation.^[^ ^33^ ^]^

To confirm that encapsulated MSCs were mechanosensitive and capable of responding to applied pressure prior to differentiation studies, we first assessed YAP localization. As a key mechanotransducer, YAP relocates to the nucleus in response to mechanical cues, initiating gene expression programs linked to osteogenesis.^[^ ^34^ ^]^ Therefore, tracking YAP localization offers valuable insight into the mechanosensitivity of encapsulated MSCs and their potential differentiation trajectory under mechanical stimulation. YAP immunostaining was performed on days 0, 7, 14, and 21 to monitor its nuclear translocation over time. The resulting images showed a clear trend of increased nuclear localization in the dynamically cultured group compared to static controls (Figure 3c). Quantification of the nuclear‐to‐cytoplasmic YAP ratio further confirmed this effect; pressure‐stimulated encapsulated MSCs exhibited consistently higher nuclear YAP levels throughout the study (Figure 3d). Statistical analysis showed that this difference was significant on days 7 and 14, indicating a mechanotransductive response to applied pressure. However, by day 21, the difference was no longer significant, with a slight decrease in nuclear YAP levels under dynamic conditions. Although continuous mechanical stimulation is generally associated with sustained YAP nuclear localization,^[^ ^35^ ^]^ our findings suggest a temporal regulation of YAP activity. This decline in nuclear YAP may reflect a shift in YAP's role: while nuclear YAP is essential for early osteogenic lineage commitment, its sustained activity may inhibit terminal osteoblast differentiation.^[^ ^36^ ^]^ Thus, the observed decrease in nuclear YAP by day 21 may represent a natural progression of differentiation, wherein YAP downregulation facilitates maturation. However, long‐term studies investigating YAP localization under sustained mechanical cues remain limited, and further work is needed to clarify this dynamic regulation.^[^ ^37^ ^]^ These findings demonstrate that encapsulated MSCs sensed and responded to applied pressure through YAP‐mediated mechanotransduction, initiating transcriptomic changes associated with differentiation.^[^ ^38^ ^]^

### 2.3 Induction of Early Osteogenic Markers under Dynamic Stimulation

After the confirmation of a mechanosensitive response, we evaluated whether MSCs could successfully undergo osteogenic differentiation within the soft microgel environment. MSC differentiation is a highly complex biological process governed by multiple factors, with substrate stiffness playing a crucial role.^[^ ^39^ ^]^ In vivo, osteoblasts reside within the rigid bone matrix, which provides a mechanically stiff environment.^[^ ^40^ ^]^ Similarly, in vitro studies have shown that MSCs preferentially differentiate into osteoblasts when cultured on rigid substrates.^[^ ^35^ ^]^ This phenomenon is well documented, as increasing substrate stiffness alone has been demonstrated to promote osteogenic marker expression.^[^ ^39^, ^40^, ^41^ ^]^ In contrast, alginate microgels are inherently soft due to their high‐water content. Although their stiffness can be tuned through polymer concentration and crosslinking density, they typically remain in the range of just a few kPa,^[^ ^42^, ^43^, ^44^ ^]^ which is considerably softer than the 11–30 kPa range often associated with osteogenesis in 3D culture systems.^[^ ^44^, ^45^ ^]^

To confirm the mechanical properties of our biomaterial system, we measured the stiffness of microgels under both standard and osteogenic culture conditions. Nanoindentation confirmed that uncoated alginate microgels exhibited effective Young's moduli below 1 kPa in both Dulbecco's Modified Eagle Medium (DMEM) and osteogenic medium (OM) (Figure S5, Supporting Information, top). After APA and calcium coating, stiffness significantly increased to 1.5 ± 0.8 kPa in DMEM and 2.4 ± 1.8 kPa in OM. We assessed the viscoelastic behavior of the microgels using dynamic mechanical analysis (DMA), as time‐dependent mechanical properties are increasingly recognized as critical regulators of stem cell fate.^[^ ^46^ ^]^ Qualitative assessment was performed by measuring the ratio of loss (G″) to storage (G′) moduli (tan δ) at 2 and 10 Hz to capture frequency‐dependent mechanical responses (Figure S5, Supporting Information, bottom). In osteogenic media, coated microgels displayed moderate tan δ values between ≈0.2 and 0.4, confirming the viscoelastic character of our microgel system.^[^ ^47^ ^]^ Prior studies have reported enhanced osteogenesis in fast‐relaxing viscoelastic hydrogels with significantly higher initial stiffness (e.g., ≈17 kPa)^[^ ^46^ ^]^; our microgels remain in the low kPa range. While this is below the threshold typically associated with robust osteogenic commitment (11–30 kPa), it falls within a regime where mechanosensitive responses have been observed under dynamic stimulation. Recent studies have shown that even ultrasoft hydrogels with moduli as low as ≈70 Pa can support osteogenesis when subjected to pulsatile pressure, suggesting that appropriate mechanical cues can compensate for limited matrix stiffness.^[^ ^48^ ^]^ Therefore, it was essential to first determine whether osteogenic differentiation could occur in our coated microgels, which fall into this soft range before applying mechanical actuation.

To this end, microgels were cultured in osteogenic induction media for 3 weeks immediately following the microgel modification process. After 1 week of culture, the cells and microgels began to aggregate, microgel shape was distorted, and extracellular matrix (ECM) production further reinforced their attachment (Figure S6a, Supporting Information). This aggregation markedly reduced the number of individually encapsulated cells available for analysis. Consequently, each visible cluster was treated as a single analysis unit, and the average fluorescence intensity was measured per aggregate (Figure S6b, Supporting Information).

Cell viability analysis showed that MSC survival was maintained at around 70% over 3 weeks (Figure S7, Supporting Information). The observed moderate cell viability likely reflects differentiation‐associated stress responses, as osteogenic commitment requires cell cycle exit and metabolic reprogramming.^[^ ^49^ ^]^ Throughout this period, cell size and nucleus size were also monitored to be able to compare them to dynamic and static culture conditions. MSCs and osteoblasts also exhibit distinct size differences, making cell and nucleus size a quantifiable parameter over the 3 week period. Notably, MSCs and osteoblasts display distinct size profiles, making these parameters useful indicators of differentiation. MSC size range is typically from 13–19 μm in diameter,^[^ ^50^ ^]^ while osteoblasts are larger, ≈20–30 μm.^[^ ^51^ ^]^ In contrast, MSC nuclei is on average ≈10 μm, compared to ≈12 μm in preosteoblasts.^[^ ^33^, ^51^ ^]^ Cell diameter showed fluctuations over time but no significant change between day 0 and day 21 (Figure S7, Supporting Information). These variations likely reflect the dynamic behavior of MSCs during proliferation and differentiation, as cell size is known to vary with cell cycle progression.^[^ ^52^, ^53^ ^]^ Additionally, physical confinement within the microgels (≤30 μm) may have influenced cellular morphology, as MSCs are sensitive to spatial constraints.^[^ ^29^ ^]^ Nuclear diameter, however, increased over the culture period (Figure S7, Supporting Information), aligning with previous findings that preosteoblasts exhibit larger nuclei than MSCs or fully differentiated osteoblasts.^[^ ^29^, ^54^ ^]^

**Figure 4:**
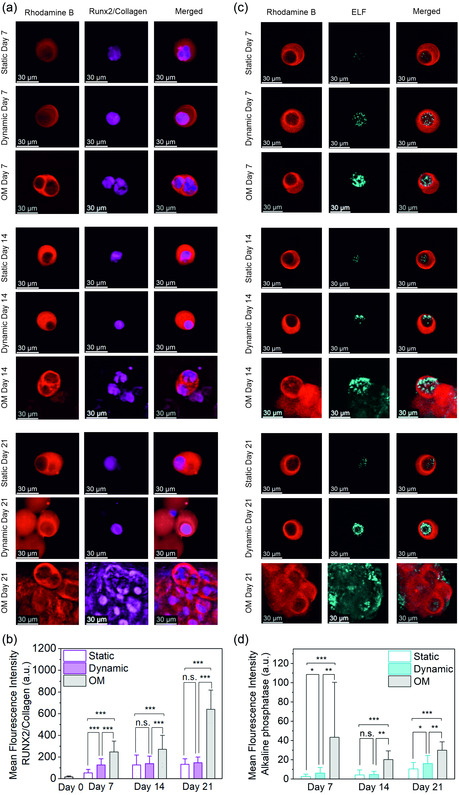
RUNX2/COL1 localization and ALP activity during osteogenic differentiation under static, dynamic, and OM conditions. a) Representative fluorescence microscopy images showing RUNX2/COL1 staining in cells cultured in static, dynamic, or OM conditions at days 7, 14, and 21. Rhodamine B (red) stains the microgels, and RUNX2/COL1 (magenta) shows nuclear or cytoplasmic localization. b) Quantification of mean RUNX2/COL1 fluorescence intensity. (*n* = 30 per group, Kruskal–Wallis test followed by Dunn's post hoc test). c) Representative fluorescence microscopy images showing ELF staining (cyan) indicating ALP activity in cells cultured in static, dynamic, and OM conditions at days 7, 14, and 21. d) Quantitative analysis of ALP fluorescence. (*n* = 30 per static and dynamic, *n* = 10 for OM group, Kruskal–Wallis test followed by Dunn's post hoc test). n.s. indicates not significant; *p < 0.05, **p < 0.01, ***p < 0.001. Error bars represent standard deviation.

To confirm osteogenic lineage commitment at the molecular level, RUNX2 and collagen type I (COL1) antibody staining were performed. RUNX2, the key transcription factor governing osteoblast differentiation, was selected for its central role in guiding MSCs toward the osteogenic lineage. As an early osteogenic marker, RUNX2 directly regulates the expression of key osteoblast‐specific genes, including osteocalcin, ALP, and collagen type I.^[^ ^55^, ^56^ ^]^ In osteogenic media‐induced samples, RUNX2 staining progressively increased around the nuclear region from day 0 to day 14, whereas by day 21, RUNX2 localization shifted predominantly to the cytoplasm (Figure S8, Supporting Information, **Figure** **4**a). A similar trend was observed in the dynamic culture group maintained in DMEM (Figure 4a). This behavior aligns with the known role of RUNX2, it remains largely cytoplasmic when inactive but translocates to the nucleus within 5 days upon osteogenic induction, where it regulates early osteogenic gene expression.^[^ ^57^, ^58^ ^]^ As differentiation progresses, RUNX2 activity decreases, leading to its redistribution and reduced nuclear presence.^[^ ^57^, ^58^ ^]^ Notably, under dynamic conditions in DMEM, overall RUNX2 expression was higher compared to the static group, as evidenced by stronger fluorescence intensities (Figure 4b). Interestingly, both groups exhibited increased expression relative to day 0, which might be attributed to the static 3D matrix properties, where stiffness alone can influence differentiation.^[^ ^57^ ^]^ Quantitative analysis of mean fluorescence intensities confirmed consistently higher RUNX2 expression in the dynamic culture group; however, statistical significance was only observed on day 7. After this point, RUNX2 expression did not significantly differ between groups, which might align with its role as an early osteogenic marker that declines as differentiation progresses.^[^ ^57^, ^58^, ^59^ ^]^

Following RUNX2 activation, osteogenic progression is typically marked by the upregulation of functional markers such as ALP.^[^ ^60^ ^]^ To investigate this transition, we assessed ALP activity over the same time course. ELF staining revealed stronger ALP fluorescence in dynamic culture groups at all time points, with particularly prominent expression observed on day 21 (Figure 4c). ALP signal intensity increased steadily throughout the 3 week period under pressure stimulation, whereas the control group showed only a modest rise. Quantitative analysis confirmed this trend, with statistically significant differences between dynamic and static groups on days 7 and 21 (Figure 4d). Interestingly, by day 21, the static culture group also exhibited elevated ALP activity, which may reflect partial osteogenic commitment induced by static mechanical cues within the 3D matrix, potentially supported by earlier observations of nuclear YAP translocation.

These findings, supported by the inclusion of the OM control group, enable a direct comparison between mechanical and biochemical routes of osteogenic induction. While osteogenic medium triggered earlier and stronger activation of early differentiation markers such as RUNX2 and ALP, pressure stimulation alone was sufficient to induce a similar sequence of gene expression with a slightly delayed onset. The OM group validates that the pattern of osteogenic marker expression observed under pressure reflects a mechanistically similar, though delayed, differentiation pathway, ultimately confirming the sufficiency of mechanical stimulation alone to trigger osteogenesis in 3D.

These results reinforce the ability of mechanical pressure to promote early‐to‐mid stage osteogenesis in soft environments, even in the absence of additional biochemical factors. Given the established role of ALP in supporting matrix deposition, we next examined whether these functional changes were accompanied by alterations in extracellular matrix synthesis and early mineralization, both of which are essential hallmarks of osteogenic progression.

### 2.4 Matrix Synthesis and Mineralization Indicate Osteogenic Commitment

After confirming the expression of early signs of differentiation in the pressure‐receiving groups, we proceeded to investigate mineralization and ECM synthesis, processes associated with midstage differentiation. As an initial step, mineralization was evaluated in 2D‐cultured MSCs treated with osteogenic medium under static and dynamic conditions. In 2D cultures, black, opaque regions indicative of calcium accumulation were observed more prominently in pressure‐treated samples. Alizarin Red S staining further confirmed increased calcium deposition under dynamic conditions compared to static controls (Figure S9, Supporting Information). Building on these observations, we next monitored mineralization progression in 3D microgel cultures over a 3 week period using bright‐field microscopy. In samples treated with osteogenic induction media, early signs of mineral deposition were evident as early as day 9, observed as dark, opaque accumulations within the microgel matrix (Figure S10, Supporting Information, **Figure** **5**a). Furthermore, 2D controls of this mineralization continued to increase steadily through Day 21. Similarly, dynamic culture samples began to exhibit blackish, opaque structures around day 14, which became more pronounced by day 21, particularly around the cell surfaces. The increased mineralization observed in dynamic group supports the successful commitment of encapsulated MSCs toward the osteogenic lineage, even within the mechanically soft microgel environment.

**Figure 5:**
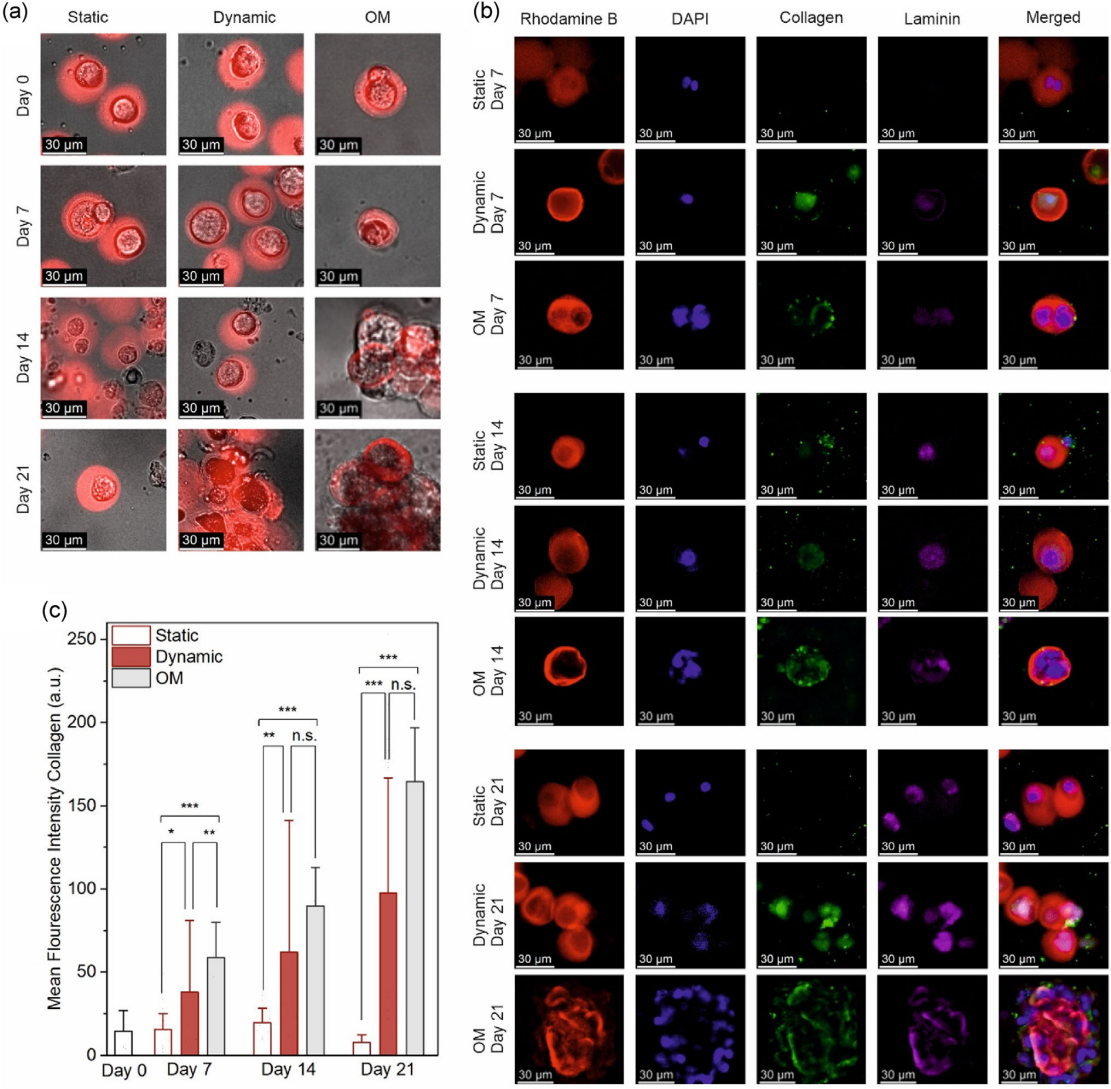
Assessment of mineralization and ECM synthesis in encapsulated MSCs under static, dynamic, and OM conditions. a) Bright‐field images of encapsulated MSCs at days 0, 7, 14, and 21. b) Immunofluorescence staining of collagen type I (green) and laminin (magenta), along with Rhodamine B (red) for hydrogel structure and DAPI (blue) for nuclei, at the indicated time points. c) Quantification of mean fluorescence intensity of collagen type I (COL1) over a 21 day period. (*n* = 30 per static and dynamic, *n* = 10 for OM group, Kruskal–Wallis test followed by Dunn's post hoc test). n.s. indicates not significant; *p < 0.05, **p < 0.01, ***p < 0.001. Error bars represent standard deviation.

We next investigated ECM synthesis, which plays a pivotal role in guiding and stabilizing mineral deposition during the later stages of osteogenic differentiation. ECM synthesis was quantified using antibody staining to monitor its progression over the 3 week period. Collagen type I (COL1) was chosen as the primary marker for ECM production, as it constitutes ≈90% of the bone ECM.^[^ ^61^ ^]^ Specifically, the deposition of collagen type I marks the transition from undifferentiated MSCs to bone‐forming osteoblasts.^[^ ^62^ ^]^ Thus, the presence and accumulation of collagen type I serves as a reliable indicator of osteogenic differentiation. In addition to collagen, laminin was selected as a marker to visualize ECM synthesis. Although present to a lesser extent in the bone ECM, laminin plays a crucial regulatory role in promoting MSC commitment to the osteogenic lineage. Elevated laminin expression has been associated with the upregulation of key osteogenic markers, such as osteocalcin, calcium, and RUNX2, suggesting that laminin contributes to the enhancement of osteogenic differentiation.^[^ ^63^ ^]^ Antibody staining of collagen type I and laminin over the 3 week period (Figure 5b) showed consistently higher fluorescence intensities of both markers in dynamic culture conditions throughout all time points, with the most pronounced differences observed on day 21. On this day, the static group showed minimal collagen and laminin staining, whereas the dynamic group exhibited substantial accumulation of both ECM proteins. For quantitative analysis, the fluorescence intensity of collagen type I was specifically evaluated due to its dominant role in extracellular matrix formation (Figure 5c). Collagen type I synthesis steadily increased throughout the three weeks in dynamic culture conditions, with statistically significant differences observed at each time point compared to the static group. In contrast, collagen levels in the static encapsulated MSCs remained largely unchanged throughout the study relative to Day 0.

To contextualize these outcomes, we compared them with an additional group cultured in osteogenic medium. While matrix synthesis and mineralization initiated earlier under biochemical induction, the overall levels achieved by day 21 in the pressure‐stimulated group were comparable. This comparison shows that the effects of hydrostatic pressure are not incidental but part of a structured differentiation process, capable of supporting late‐stage osteogenic maturation in the absence of soluble cues.

The observed increase in collagen fluorescence is a robust indicator of osteogenesis, as ECM secretion is a definitive marker of MSC differentiation toward osteoblasts.^[^ ^64^ ^]^ The statistical difference beginning on day 7 also suggests that various cells exhibited osteogenic behavior on the first week of pressure stimulation. This observation aligns with existing literature, as numerous studies investigating pressure‐induced differentiation have reported increased osteogenic marker expression within only a few days.^[^ ^20^, ^50^ ^]^ As noted previously, ECM production is a hallmark of mid‐stage differentiation, suggesting that on day 21 pressure‐stimulated encapsulated MSCs had likely committed to the differentiation, with ECM deposition advancing toward the formation of a fully mineralized bone matrix.^[^ ^64^ ^]^ The elevated mean collagen expression indicates that many cells are actively undergoing mechanotransduction and secreting ECM, signifying osteogenic commitment in only pressure‐treated samples without any induction media.

During extended culture in OM, microgels spontaneously aggregated into ECM‐rich clusters, which made it impossible to resolve individual cells for quantification. Although the core microgel structures remained partially preserved, matrix bridging and intercellular connections were frequently observed from day 14 onward (Figure S6, Supporting Information). To address this, we analyzed each visible cluster as a single unit by measuring average fluorescence intensity per aggregate. This approach enabled a representative assessment of marker expression trends despite architectural complexity. We acknowledge that microgel aggregation in the OM condition may itself reinforce osteogenic signaling. It is known that even in the absence of biochemical cues, MSC spheroid formation can trigger osteogenesis through increased compaction, matrix‐mediated feedback, and cell–cell proximity.^[^ ^65^ ^]^ Therefore, we interpret the OM group not as a direct comparator to the pressure‐treated groups, but rather as a biologically distinct positive control demonstrating the osteogenic trajectory under combined biochemical and physical clustering effects. In contrast, the pressure‐treated system maintains near single‐cell resolution and isolates the effects of mechanical stimulation alone. In our soft, viscoelastic microgels, cyclic hydrostatic pressure acted as the decisive input for osteogenesis, achieving outcomes typically attributed to high matrix modulus while remaining compatible with soft, injectable systems.

## 3 Conclusion

This work introduces a dynamic microgel platform capable of directing osteogenic differentiation of singly encapsulated MSCs through mechanical stimulation alone. The APA and calcium‐based coating strategy ensured long‐term single‐cell encapsulation and viability, enabling high‐resolution, time‐resolved analysis of cell behavior under confinement. By applying cyclic hydrostatic pressure within soft, viscoelastic microgels, we show that mechanical cues are sufficient to activate mechanotransduction in encapsulated stem cells, induce osteogenic differentiation, measured by early differentiation markers RUNX2 and ALP, and promote matrix deposition and mineralization. Our approach enabled osteogenic differentiation achieved in the absence of biochemical induction, emphasizing the potential of force‐driven approaches for guiding stem cell fate. Notably, these outcomes were obtained in viscoelastic, soft single‐cell microgels, indicating that cyclic hydrostatic pressure can substitute for the high static stiffness and is inherently compatible with soft, injectable carriers. This platform lays the groundwork for scalable and programmable single‐cell therapies, providing a versatile tool to investigate the interplay between mechanical stimulation and lineage commitment within soft, injectable systems, where microgels function as both cell carriers and dynamic culture environments.

While mouse MSCs were used here to establish feasibility, validation with human MSCs will be an essential next step to confirm the clinical and translational potential of the system. Future studies will therefore focus on human MSC differentiation and on tuning the mechanical parameters and pressure profiles specifically for human MSCs. New material systems will be investigated to study the combinatorial effects of stiffness and viscoelasticity together with hydrostatic pressure. In addition, transcriptional analyses will be performed to validate the expression of key osteogenic markers and to further confirm the differentiation trajectory observed under mechanical stimulation. Efforts will also explore directing differentiation toward alternative lineages, such as chondrogenesis, to precisely map commitment trajectories. Mechanically active microgels hold significant promise as powerful, modular platforms for engineering stem cell fate with high precision, paving the way toward next‐generation regenerative therapies that are both scalable and aligned with natural biological processes.

## 4 Experimental Section

4.1

4.1.1

##### Fabrication of Pressure Device

A standard 15 mL conical tube was adapted to function as a sealed pressure vessel. The interior was coated with 3% Pluronic to minimize cell adhesion, rinsed with phosphate‐buffered saline (PBS), and maintained under sterile conditions throughout the preparation process. The cap was modified with a centrally drilled port, into which a wide‐diameter syringe filter (Fisherbrand, PES, 33 mm, 0.22 μm pore size) was mounted and sealed. From the filter outlet, airflow passed through a male‐to‐male Luer‐lock connector (Combifix Adapter, B. Braun) into a female/male extension tubing (Extension Line Type: Heidelberger, B. Braun), which then connected to a second identical syringe filter positioned upstream of the pressure chamber to maintain sterility from the incoming compressed air. All junctions were secured with Luer‐lock fittings to ensure airtight integrity and minimize pressure loss during repeated cycling. The syringe filter was then connected directly to the pressure controller (OB1 MK3+ Pressure Controller, Elveflow). To enable pressure monitoring, a hole was drilled at the edge of the pressure tube, where a sensor device (MPS Pressure Sensor, Elveflow) was connected (Figure S1d,e, Supporting Information). The pressure sensor was connected to the pressure controller, allowing the system pressure to be continuously monitored and recorded via a computer interface.

##### Fabrication of Microfluidic Device

Microfluidic devices were fabricated using standard soft lithography techniques. Briefly, a device master was produced by spin‐coating a 25 μm thick layer of SU‐83 050 photoresist (Kayaku Advanced Materials) onto a clean silicon wafer. The coated wafer was exposed to UV light at 250 μJ cm^−2^ using a μMLA maskless aligner (Heidelberg Instruments), followed by postbaking and development according to the manufacturer's protocol. After fabrication of the SU‐8 master, a degassed mixture of polydimethylsiloxane (PDMS; Sylgard 184, VWR) and crosslinker (10:1 ratio) was poured onto the master and cured at 65 °C for a minimum of 1 h. Once cured, the PDMS layer was peeled off, and inlet/outlet ports were created using a 1 mm biopsy punch. The PDMS slab was bonded to a glass slide via oxygen plasma treatment (Piezobrush PZ3) applied to both surfaces. To increase hydrophobicity, channels were treated with RainX and dried overnight at 65 °C. Prior to use, the chips were sterilized by immersion in 70% ethanol and air‐dried under sterile conditions.

##### Alginate Preparation and Functionalization

High molecular weight alginate (I‐1 G, KIMICA) was used to prepare the microgels. To prepare functionalized alginate, carbodiimide‐mediated coupling was used to attach either an RGD peptide or Rhodamine B. Alginate was dissolved in MES buffer at 1% (w/v) and reacted with the integrin‐binding peptide GGGGRGDSP (Peptide 2.0) at a degree of substitution (DS) of 20, or with Rhodamine B Lissamine (Thermo Fisher Scientific) at a DS of 2. EDC and sulfo‐NHS (Thermo Fisher Scientific) were added at a molar ratio of 2:1 to activate the carboxyl groups. The reactions proceeded for 20 h at room temperature. The purification steps applied to unmodified alginate were also used for both RGD‐ and Rhodamine B‐functionalized variants, which were subsequently lyophilized and stored at −20 °C until use. All reagents were obtained from Sigma–Aldrich unless otherwise specified.

##### Cell Encapsulation and Culture

Mouse bone marrow stromal cells (D1 ORL UVA; ATCC) were cultured in high‐glucose DMEM (Gibco, US) supplemented with 10% fetal bovine serum (FBS; Gibco, US) and 1% penicillin–streptomycin (Gibco, US). Cells were maintained under subconfluent conditions. For encapsulation, cells were processed according to previously established protocols.^[^ ^21^, ^26^, ^66^ ^]^ Briefly, D1 cells were trypsinized and washed with 10 mg mL^−1^ CaCO_3_ nanoparticles (Essence 70PCC) to promote surface adsorption. The cell suspension was mixed with alginate to yield a final alginate concentration of 1 wt% and a cell density of 25 million cells mL^−1^ in the prepolymer solution. The aqueous phase consisted of both RGD‐functionalized alginate (degree of substitution, DS 20) or and Rhodamine B‐labeled alginate at a volumetric ratio of 4:1. The oil phase was prepared using 1 vol% Pico‐Surf (Sphere Fluidics), 0.04 vol% glacial acetic acid (Sigma–Aldrich), and Novec HFE‐7500 fluorinated oil. The aqueous and oil phases were introduced into a microfluidic chip via separate inlets and co‐flowed using a syringe pump (Darwin Microfluidics) at a rate of 1.7 μL min^−1^. The emulsion was collected into pipette tips every 30 min and processed in batches. During encapsulation, both the microfluidic chip and collection tips were agitated on an orbital shaker (Fisherbrand Multi‐Platform Shaker) at 600 rpm to promote central cell positioning within the forming microgels. Postencapsulation, samples were incubated on ice for 15 min, followed by demulsification using 10% 1 H,1 H,2 H,2 H‐Perfluoro‐1‐octanol.

For surface coating, microgels were treated using an adapted protocol based on previously established methods.^[^ ^27^, ^66^ ^]^ Microgels were first transferred to 15 mL conical tubes and centrifuged at 270 rcf for 5 min. The supernatant was discarded, and microgels were resuspended in 2 mL of 0.01 mg mL^−1^ PLL and incubated for 5 min. Following another centrifugation step, the PLL solution was removed, and the microgels were suspended in 0.001% alginate prepared in complete DMEM. This mixture was incubated for an additional 5 min to complete the layering process. To stabilize the coating, the microgels were washed with HEPES buffer (25 mM HEPES, 130 mM NaCl, 1.8 mM CaCl_2_). For calcium crosslinking, 30 mM CaCl_2_ in HEPES buffer was added to microgels and incubated for 7 min. Excess calcium was removed by diluting the mixture with 10 mL of a 3:2 mixture of complete DMEM and 1 × PBS (Gibco, US). Microgels were centrifuged again at 270 rcf for 5 min, and half of the solution was removed.

Following the modification, microgels were cultured in 3% Pluronic‐coated 6‐well plates. Wells were coated with 3 mL of 1% Pluronic for 20 min, washed with HEPES buffer, and filled with 3 mL of medium, keeping the cell concentration at 2–3 × 10^5^ cells mL^−1^. Plates were incubated on a 3D nutating mixer (GyroMini nutating mixer) in a standard cell incubator to minimize agglomeration. The microgels containing D1 cells were statically cultured in either complete DMEM prepared from powder or in osteogenic induction medium prepared using the Gibco StemPro Osteogenesis Differentiation Kit (Thermo Fisher Scientific). Complete DMEM for encapsulated cell culture was prepared manually from Gibco DMEM powder (high glucose, pyruvate) by dissolving the powder in 1 L of distilled water, supplementing with 10% FBS and 1% P/S, and buffering with 3.7 g L^−1^ sodium bicarbonate. The pH was adjusted to 7.4 by adding the required amount of HCl.

For dynamic culture conditions, cells were transferred daily into a 15 mL conical pressure device containing 6 mL of culture medium with ≈1 × 10^6^ cells in microgels and 9 mL of headspace and subjected to a pressure of 200 kPa at 0.5 Hz for 30 min over a 3 week period.

##### Cell Staining and Quantification

Cell viability of encapsulated cells was assessed using 2 μM Calcein‐AM and 4 μM Ethidium homodimer‐1 (Thermo Fisher Scientific, Invitrogen).

Prior to immunostaining, 96‐well plates were coated with PLL and incubated for 1 h. Excess PLL was removed, and microgels were transferred into the wells. Samples were washed 3 times with HEPES buffer and fixed in 4% paraformaldehyde for 30 min at room temperature. Following fixation, microgels were washed again and stored in HEPES buffer at 4 °C until further staining. The samples were treated with 0.3% Triton‐X in HEPES buffer and blocking was performed using 10% goat serum to reduce nonspecific binding. Immunostaining was performed using various antibodies, including RUNX2 (Invitrogen, #PA5‐86 506), COL1A2 (Invitrogen, #PA5‐96 426), YAP1 Rabbit Polyclonal Antibody (Invitrogen #PA1‐46 189), laminin (Invitrogen, #PA5‐22 901), and DAPI. Unconjugated primary antibodies were incubated overnight at 4 °C in 2% goat serum, followed by secondary antibody staining with Alexa Fluor 488 or 647 conjugates (Abcam, ab150077 and ab150079, respectively). Laminin conjugated antibody was added during the secondary antibody step. DAPI (1 μg mL^−1^) was used as a nuclear stain. Samples were mounted in HEPES buffer and imaged using appropriate filter sets.

Endogenous phosphatase activity was assessed using ELF 97 Phosphatase Substrate (Thermo Fisher Scientific). Fixed samples were permeabilized with 0.3% Tween‐20 in HEPES buffer for 10 min, rinsed, and incubated in the ELF 97 solution (1:30 dilution, filtered through 0.2 μm) for 2 min. Staining was terminated by triple washing with HEPES buffer. Imaging was performed using a DAPI channel.

Fluorescence imaging was performed using an inverted fluorescence microscope (DMi8 Series, Leica Microsystems, Germany). Quantification of live and dead cells was conducted in ImageJ, using a sample size of 100 cells per group, repeated across three independent replicates. For YAP quantification, regions of interest (ROIs) corresponding to the nucleus and cytoplasm were defined, and the nuclear YAP ratio was calculated by dividing nuclear fluorescence intensity by cytoplasmic intensity. For all other stains, fluorescence quantification was performed by selecting an ROI encompassing the entire cell. In OM‐treated conditions at days 7, 14, and 21, cell‐laden microgels exhibited spontaneous aggregation due to matrix deposition. In these cases, single‐cell segmentation was not feasible. Therefore, we manually defined ROIs around each aggregate and measured the mean fluorescence intensity of the entire cluster after background subtraction (Figure S6b, Supporting Information). Measurements were analyzed using Leica Microsystems Suite X software. A sample size of 30 cells per group was used for all antibody‐based quantification, 10 aggregates were analyzed for OM‐treated groups.

##### Nanoindentation

Mechanical characterization of the microgels was conducted using the Piuma Chiaro nanoindenter system (Optics11). A spherical cantilever probe with a spring constant of 0.48 N m^−1^ and a tip radius of 10.5 μm was employed. Indentation measurements were performed to a depth of 1000 nm from the microgel surface. The effective Young's modulus was determined based on a linear Hertzian contact model. Prior to measurement, the probe was calibrated, and all indentations were carried out in either DMEM or OM. For DMA, a fixed oscillatory amplitude of 300 nm was applied at two frequencies (2 and 10 Hz) to evaluate frequency‐dependent mechanical responses. For each condition and frequency, five repetitions were conducted with a 2 s rest interval between measurements. DataViewer V2 software (Optics11) was used for analysis.

##### Statistical Analysis

All statistical analyses were performed using OriginPro 2021b. Data were presented as mean ± standard deviation. A significance level of 0.05 was used for all tests. Normality was assessed with the Shapiro–Wilk test and homogeneity of variances with Levene's test. For comparisons between two groups, Student's *t*‐test was applied. For comparisons involving three groups, one‐way ANOVA followed by Tukey's post hoc test was used to assess statistical significance. Where the normality is rejected, Kruskal–Wallis test followed by Dunn's post hoc test was applied. Sample sizes for each analysis are provided in the corresponding figure captions. The following *p*‐values were used to indicate significance: *p < 0.05; **p < 0.01; ***p < 0.001; and ****p < 0.0001.

## Supporting Information

Supporting Information is available from the Wiley Online Library or from the author.

## Conflict of Interest

The authors declare no conflict of interest.

## Supporting information

Supplementary Material

## Data Availability

The data that support the findings of this study are available from the corresponding author upon reasonable request.
