# Supplementary material for: Hydrostatic Pressure Induces Osteogenic Differentiation of Single Stem Cells in 3D Viscoelastic Microgels
Source: Small Sci. 2025 Sep 21;5(12):e202500287. doi: 10.1002/smsc.202500287 (PMC12697899; doi:10.1002/smsc.202500287)
Supplement: Supplementary file 1 — Supplementary Material [file SMSC-5-e202500287-s001.pdf]

Supporting Information

Hydrostatic Pressure Induces Osteogenic Differentiation of Single Stem Cells in 3D Viscoelastic Microgels

*Nergishan İyisan, Fernando Rangel, Leonard Funke, Bingqiang Pan, Berna Özkale**

N. İyisan, F. Rangel, L. Funke, B. Pan, B. Özkale
Microrobotic Bioengineering Lab (MRBL), School of Computation, Information and Technology, Department of Electrical Engineering, Technical University of Munich (TUM), Hans-Piloty-Straße 1, Garching 85748, Germany
E-mail: berna.oezkale@tum.de

N. İyisan, F. Rangel, L. Funke, B. Pan, B. Özkale
Munich Institute of Robotics and Machine Intelligence, Technical University of Munich, Georg-Brauchle-Ring 60, 80992 München, Germany

N. İyisan, F. Rangel, L. Funke, B. Pan, B. Özkale
Munich Institute of Biomedical Engineering, Technical University of Munich, Boltzmannstraße 11, 85748 Garching, Germany

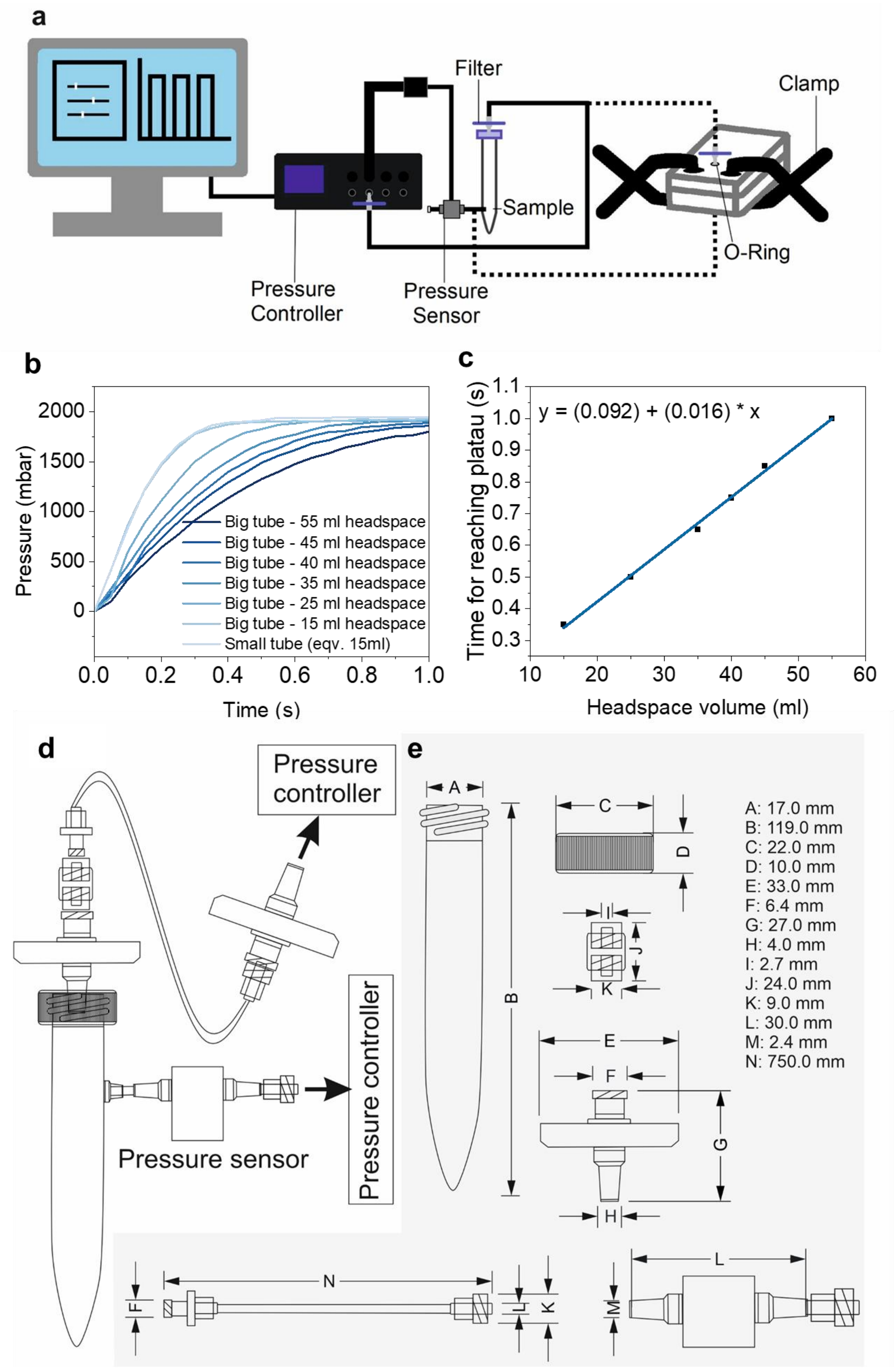
a
Filter
Clamp
Sample
O-Ring
Pressure Controller
Pressure Sensor
b
Pressure (mbar)
Time (s)
Big tube - 55 ml headspace
Big tube - 45 ml headspace
Big tube - 40 ml headspace
Big tube - 35 ml headspace
Big tube - 25 ml headspace
Big tube - 15 ml headspace
Small tube (eqv. 15ml)
c
y = (0.092) + (0.016) * x
Time for reaching platau (s)
Headspace volume (ml)
d
Pressure controller
Pressure sensor
Pressure controller
e
A: 17.0 mm
B: 119.0 mm
C: 22.0 mm
D: 10.0 mm
E: 33.0 mm
F: 6.4 mm
G: 27.0 mm
H: 4.0 mm
I: 2.7 mm
J: 24.0 mm
K: 9.0 mm
L: 30.0 mm
M: 2.4 mm
N: 750.0 mm

Figure S1. Schematic representation of the pressure stimulation setup. (a) The system consists of a computer-controlled pressure controller, a pressure sensor for real-time feedback, a filter to prevent contamination, and a sealed sample chamber secured with an O-ring and clamp. Two different configurations were used for pressure application: a well-plate setup (indicated by dotted lines) and a conical tube setup (indicated by solid lines), each optimized for different experimental applications. (b) In-situ pressure traces at the setpoint for conical tubes with varied headspace volumes; a 15 mL tube with matched headspace is shown for reference. (c) Time-to-setpoint versus headspace volume with linear fit. Pressure was measured using an inline sensor. (d)Detailed view of the conical tube configuration showing the pressure line connection, pressure sensor placement, and sealing components. (e) Technical drawings with dimensions of the fittings, adapters, and tube cap enabling a secure and airtight connection between the conical tube and the pressure line.

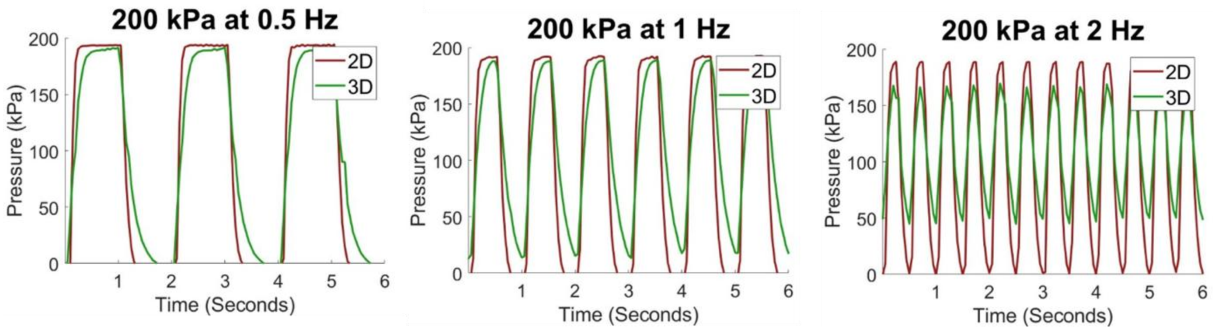

Figure S2. Pressure profile characterization of the stimulation system under different frequency settings. Representative pressure waveforms recorded by the integrated pressure sensor at 200 kPa with stimulation frequencies of 0.5 Hz, 1 Hz, and 2 Hz. Measurements were performed in both 2D (well-plate) and 3D (conical tube) configurations.

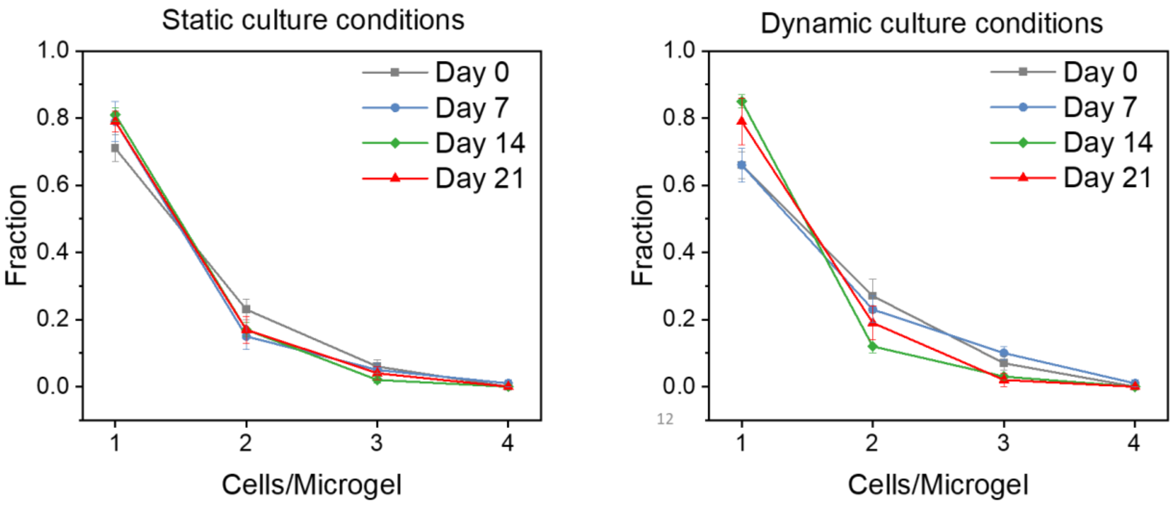

Figure S3. Encapsulated MSC proliferation within microgels over a 21-day period under static and dynamic conditions.

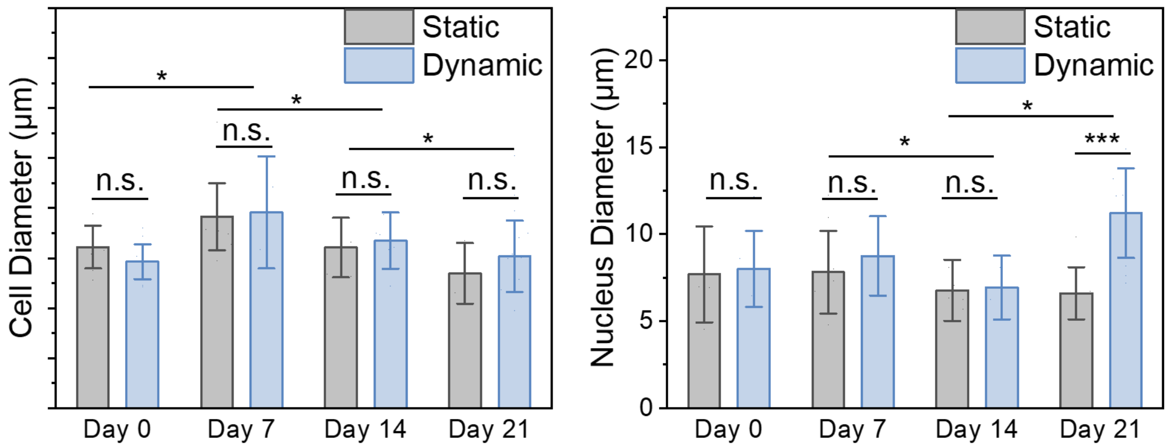

Figure S4. Changes in cell and nucleus diameter over time under static and pressure conditions. Quantification of cell (left) and nucleus (right) diameters in static and dynamic groups cultured in DMEM over 21 days (n=30). n.s. indicates not significant; $*p < 0.05$, $**p < 0.01$, $***p < 0.001$; statistical comparisons were performed using two-way ANOVA followed by Tukey's post hoc test. Error bars represent standard deviation.

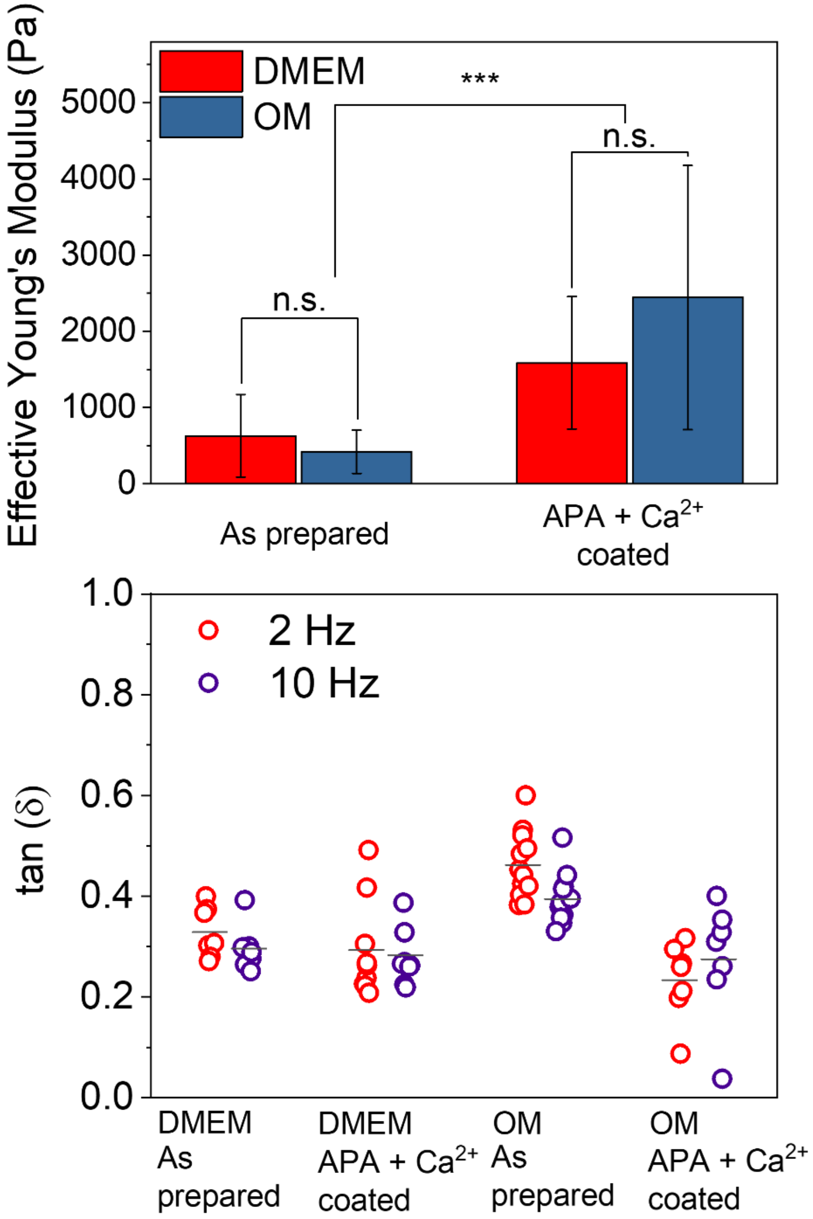

Figure S5. Mechanical characterization of microgels before (As prepared) and after APA + Ca^{2+} coating in DMEM and osteogenic induction medium (OM). (Top) Stiffness measurements of microgels (n = 10 per group, two-way ANOVA followed by Tukey's post hoc test). n.s. indicates not significant; *$p < 0.05$, **$p < 0.01$, ***$p < 0.001$. Error bars represent standard deviation. (Bottom) Dynamic mechanical analysis

(DMA) of the same groups, showing tan δ values at 2 Hz and 10 Hz. Each data point represents an individual microgel measurement.

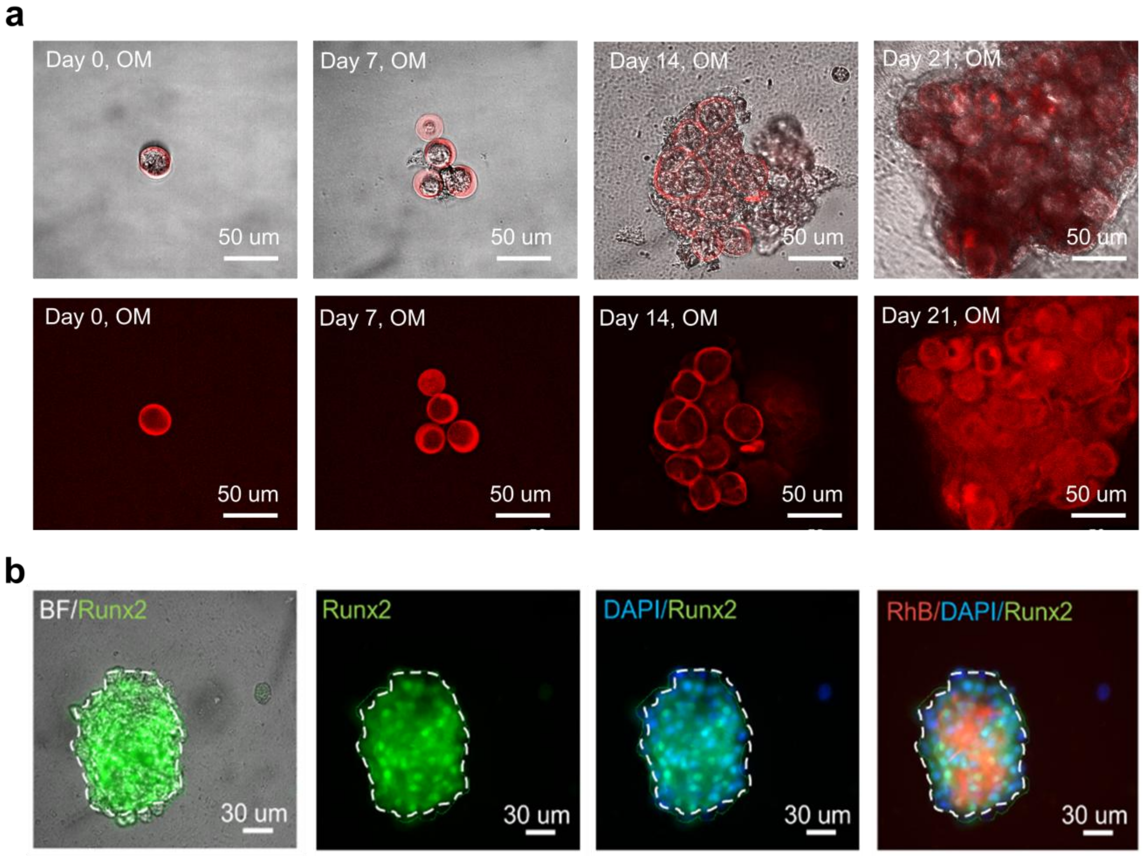

Figure S6. Osteogenic media (OM) induced aggregation. a) Time-lapse brightfield/fluorescence images showing spontaneous aggregation of cell-laden microgels during extended culture in osteogenic medium (OM). Microgels obtained as single units at Day 0 progressively formed clusters by Day 7, with more extensive aggregation and ECM-rich bridging observed by Days 14 and 21. The red signal indicates Rhodamine B. Scale bar: 50 µm. b) Example aggregate with region of interest (ROI) boundary (white dashed line). Scale bar: 30 µm.

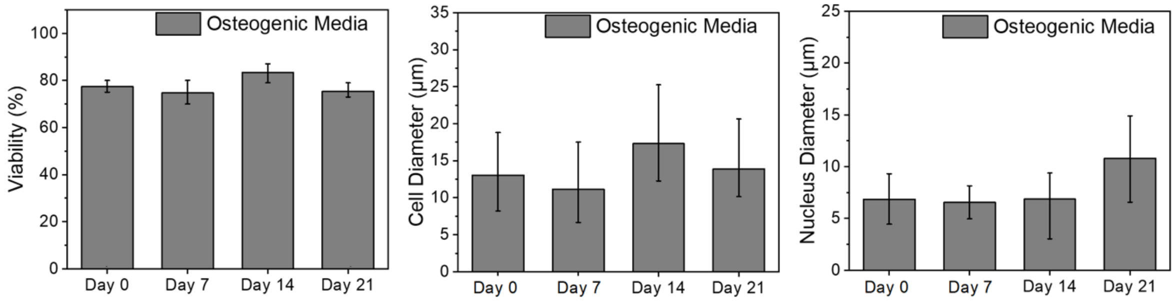

Figure S7. Representative images of encapsulated MSCs treated with osteogenic medium on day 7. Bright-field, Rhodamine B-labeled microgels (red), Collagen I immunostaining (green), and merged fluorescence channels are shown.

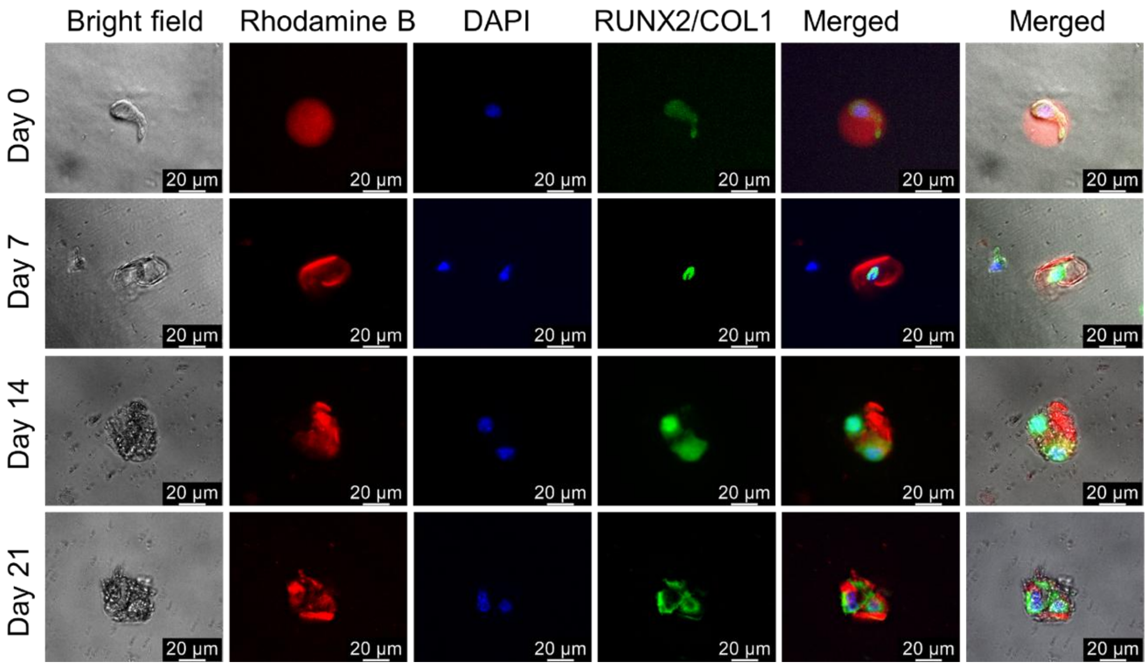

Figure S8. Temporal expression and localization of RUNX2/COLLAGEN1 during osteogenic differentiation in the samples treated with osteogenic induction medium. Representative images showing RUNX2/COLLAGEN1 expression in encapsulated cells at Days 0, 7, 14, and 21 of osteogenic induction. Bright field and Rhodamine B (red) images depict microgels. DAPI (blue) stains nuclei, and RUNX2 (green) indicates the transcription factor's localization.

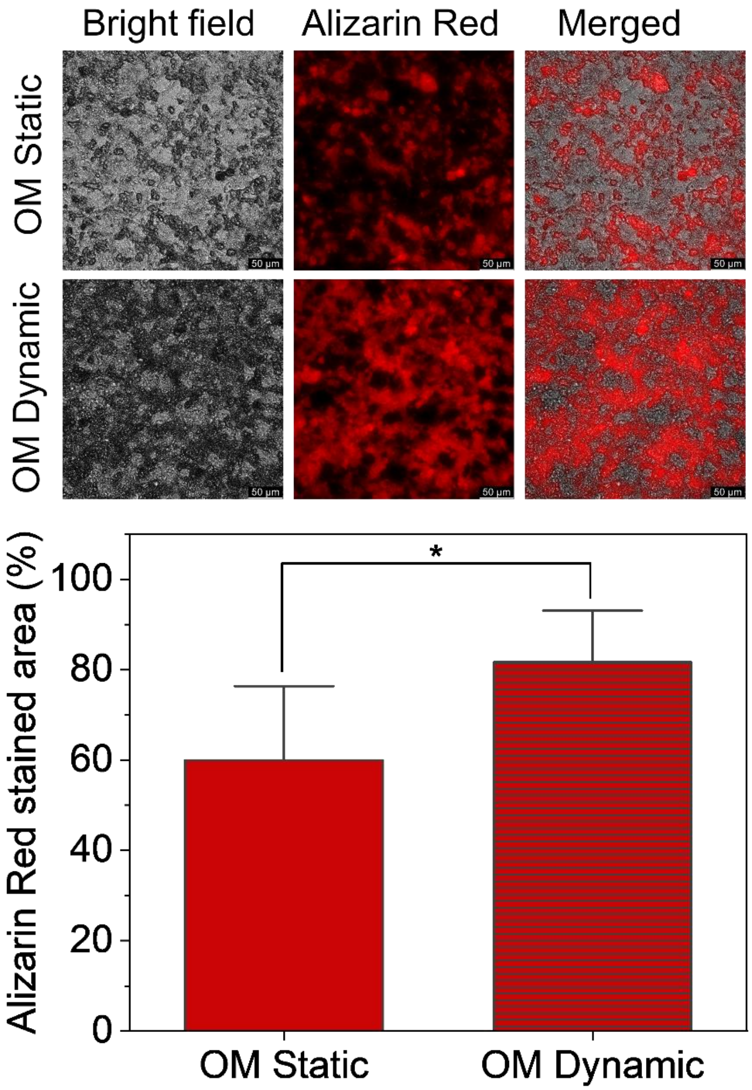

Figure S9. Representative microscopy images of 2D-cultured MSCs stained with Alizarin Red S after 21 days of culture under static osteogenic medium (OM) and osteogenic medium with cyclic pressure stimulation (OM + Pressure). Left panels show brightfield images, middle panels show fluorescence images of Alizarin Red S staining, and right panels show merged brightfield and fluorescence images. Quantification of Alizarin Red-positive area (%) is shown in the bar graph. (n=5, Student's t-test, *$p < 0.05$,). Error bars represent standard deviation.

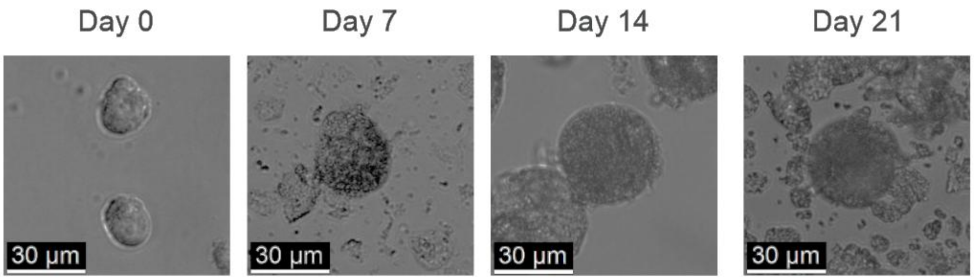

Figure S10. Brightfield images showing morphological changes and mineral deposition during osteogenic differentiation. Representative brightfield images of encapsulated cells treated with osteogenic induction medium at Days 0, 7, 14, and 21. Early signs of mineral deposition, visible as dark and dense accumulations within the microgel matrix, became evident from Day 7 and increased progressively through Day 21, indicating active matrix remodeling and mineralization.
